# Cell-free DNA levels of twins and sibling pairs indicate individuality and possible use as a personalized biomarker

**DOI:** 10.1371/journal.pone.0223470

**Published:** 2019-10-10

**Authors:** Lamyaa Alghofaili, Hannah Almubarak, Khawlah Gassem, Syed S. Islam, Serdar Coskun, Namik Kaya, Bedri Karakas

**Affiliations:** 1 Translational Cancer Research Section, Department of Molecular Oncology, King Faisal Specialist Hospital and Research Center, Riyadh, Saudi Arabia; 2 Alfaisal University Medical School, Riyadh, Saudi Arabia; 3 Department of Pathology and Laboratory Medicine, King Faisal Specialist Hospital and Research Center, Riyadh, Saudi Arabia; 4 Department of Genetics, King Faisal Specialist Hospital and Research Center, Riyadh, Saudi Arabia; CNR, ITALY

## Abstract

Cell-free DNA (cfDNA) in the human blood circulation has been under investigation since its initial observation in 1948. Plasma cfDNA is known to be significantly elevated in diseased people. Due to possible variation in the population, evaluating cfDNA as a non-invasive biomarker at disease onset alone may not be sensitive enough to accurately diagnose diseases, particularly early stage cancers on a personal level. To understand the factors that define the cfDNA levels on the personal level and for better use as a non-invasive biomarker, we isolated cfDNA from the plasma of healthy individuals with varying degrees of genetic and/or environmental similarities (monozygotic twins, dizygotic twins, sibling pairs, and unrelated individuals) as well as from patients with varying stages of breast and ovarian cancer undergoing treatment. Cell-free DNA levels were quantified by a fluorometer (ng/ml) and/or real-time PCR (copies/ml). The associations between individuals with various degrees of genetic and/or environmental similarities and their plasma cfDNA levels were evaluated. The ACE model (A = additive genetic, C = common environment, and E = specific environmental factors) was used to determine the proportion of each factor on the cfDNA levels. We found a high correlation (r = 0.77; p < 0.0001) in plasma cfDNA levels between monozygotic twins (n = 39). However, the correlation was gradually reduced to moderate (r = 0.47; p = 0.016) between dizygotic twins (n = 13) and low correlation (r = 0.28; p = 0.043) between sibling pairs (n = 26). The ACE model analysis showed that the plasma cfDNA level of a given healthy individual is influenced both by genetic and the environmental components in similar proportions (53% and 47%, respectively; A = 53%, C = 22.5%, E = 24.5%). Moreover, while age had no effect, gender significantly influenced the individual’s plasma cfDNA level. As expected, cfDNA levels were significantly higher in both breast (n = 26) (p<0.0001) and ovarian (n = 64) (p<0.0001) cancer patients compared to the healthy individuals. Our study demonstrated that both genome and environmental factors modulate the individual’s cfDNA level suggesting that its diagnostic sensitivity may be improved only if the person’s cfDNA level is known prior to disease presentation.

## Introduction

The report of cell-free DNA (cfDNA) in the blood circulation of healthy and diseased individuals precedes even the discovery of the DNA double helix [1]. Following this initial observation, the possible use of cfDNA as a non-invasive biomarker has been investigated in monitoring various disease conditions using patient’s serum/plasma as well as detecting fetal DNA in maternal blood circulation [2–6]. The source and nature of cfDNA have also been studied however many unknown aspects are still awaiting to be discovered [7,8].

Cell-free DNA is continuously released into the blood circulation and also rapidly cleared with a mean half-life of about 15 minutes [9], therefore, it is highly fragmented [10,11]. The mode by which cfDNA gets into the blood circulation is still largely unknown. However, there are two main suggested release mechanisms including the release as a result of cell death (i.e., necrosis and apoptosis) and the active secretion from healthy cells [12–14].

To date, there are no reports of plasma cfDNA solely focusing on healthy individuals except in cases to improve extraction and quantification methods [15–18]. Furthermore, estimates of cfDNA levels in disease-free individuals, which have been reported in cancer studies as control groups, show a considerable variation [19–22]. Due to this variation even among healthy individuals, the diagnostic value of cfDNA as a noninvasive biomarker on the personal level is vastly diminished. Therefore, cfDNA should be studied more thoroughly to circumvent this drawback that is associated with high variation in the population.

Attempts to use plasma/serum cfDNA levels as a non-invasive biomarker has not been successful in detecting diseases reliably especially for early stage cancers [23]. Therefore, the focus has been shifted towards more targeted approaches such as detecting somatic DNA aberrations in cell-free tumor DNA (ctDNA) in the blood circulation by digital PCR (i.e. BEAMing, ddPCR) and next generation sequencing (NGS) [24–26]. However, intratumor heterogeneity due to genomic instability, loss of heterozygosity (LOH), and clonal selection throughout tumor progression in addition to selection during therapy may cause certain mutations to be either lost or enriched [27–30]. Hence, total plasma cfDNA levels could be used along with these targeted digital detection approaches for higher sensitivities.

**Fig 1:**
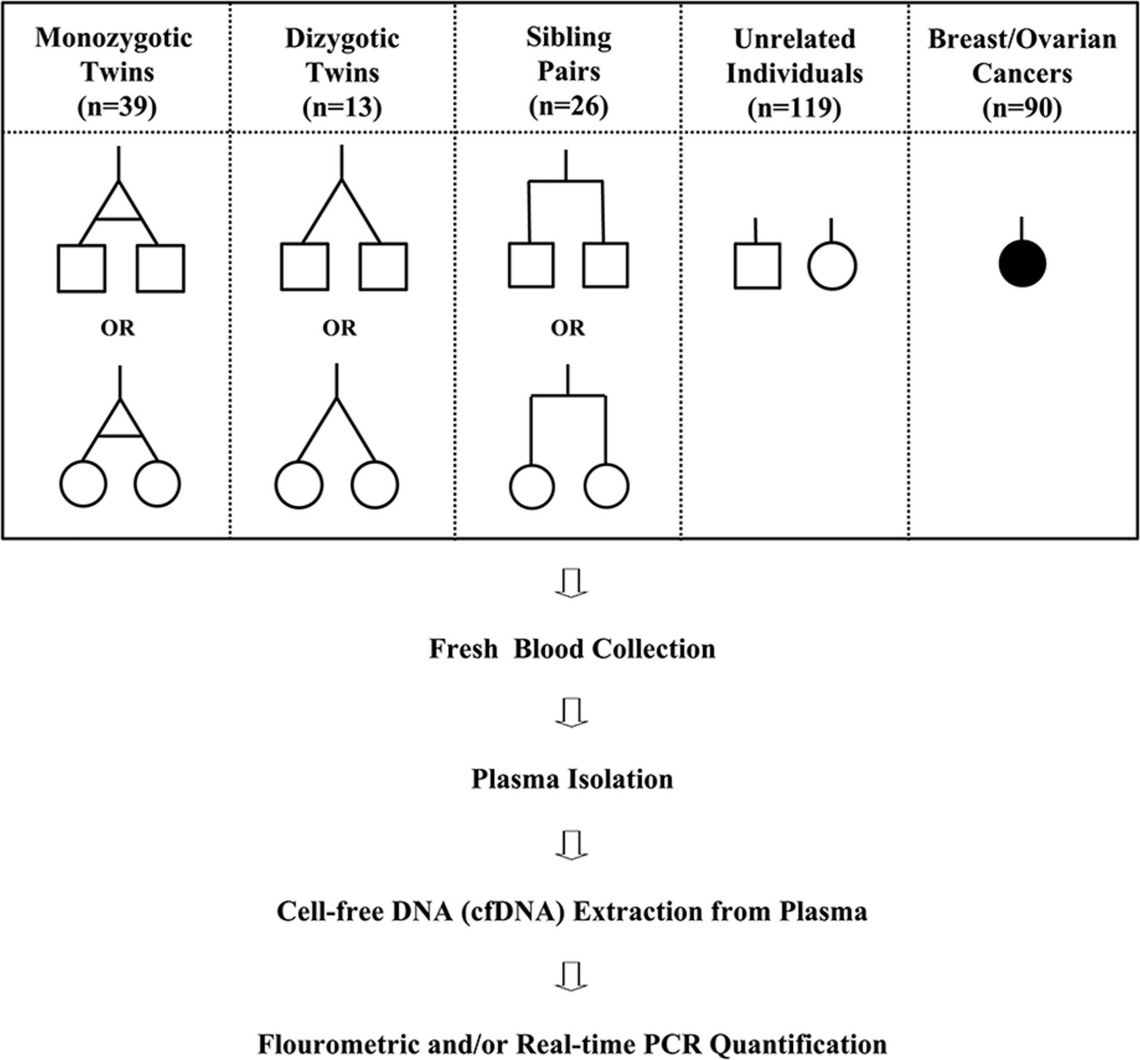
Study design and blood sample collection. The schematic illustration of experimental design showing blood sample collection from healthy individuals and cancer patients, and cell-free DNA (cfDNA) quantification steps. A total of 275 fresh blood samples were collected from monozygotic (n = 39) twins, dizygotic (n = 13) twins, sibling pairs (n = 26), genetically unrelated healthy individuals (n = 119), and a total of 90 blood samples from breast (n = 26) and ovarian cancer (n = 64) patients. Plasma was isolated from a fresh blood sample then cfDNA was extracted from plasma and quantified by a fluorometer (ng/ml) and/or by real-time quantitative PCR (copies/ml).

We believe that there are both genetic and environmental aspects defining the ultimate level and degradation status of cfDNA in the individual’s blood circulation. In this proof-of-principle study, we analyzed cfDNA isolated from a total of 365 plasma samples (monozygotic twins, dizygotic twins, sibling pairs, genetically unrelated individuals, cancer patients) (Fig 1). Plasma samples were analyzed both quantitatively and qualitatively in an attempt to determine the source of variation and estimate the relative contribution of genetic and environmental factors, which could help to be better used as a predictive and prognostic non-invasive biomarker. Although we observed similar plasma cfDNA levels between monozygotic twins, there was a substantial variation among genetically unrelated individuals.

## Materials and methods

### Ethics statement

This study was approved by the Research Ethics Committee (RAC) of the King Faisal Specialist Hospital and Research Center (KFSH&RC) (RAC#2130037). All methods have been carried out in accordance with relevant guidelines and regulations. All subjects were properly informed about the study and their written consents were obtained. For subjects who were under age 18 years, informed consents were provided by either a parent or a legal guardian.

### Study design and blood sample collections

Our study population included a total of 275 healthy female (n = 151) and male (n = 124) individuals; 39 monozygotic twins, 13 dizygotic twins, 26 sibling pairs, 119 unrelated healthy individuals, and 90 breast and ovarian cancer patients (Fig 1). All individual twins and sibling pairs were of the same gender (i.e., male/male or female/female) except in one case of a dizygotic twin being different (i.e. male/female). The overall age of the healthy subjects ranged from zero to 57 years old with a median age of 23 years. All blood samples of genetically unrelated healthy individuals were obtained from the Blood Bank of KFSH&RC whereas twins and sibling samples were collected from recruited volunteers for the study. Samples from blood bank donors were obtained as an additional tube and were immediately transferred to the laboratory for processing to avoid technical variation. Blood samples from healthy individuals were collected mostly during the afternoon hours. In addition to healthy individuals, blood samples were collected from 26 breast cancer and 64 epithelial ovarian cancer patients who had varying stages of cancer and undergoing treatment at KFSH&RC. Fresh blood samples were drawn into BD Vacutainer EDTA tubes (Cat# 366643), and immediately centrifuged at 3,000 rpm for 10 min at +4 ^o^C, and the plasma fractions were collected and re-centrifuged in a second centrifugation step for 10 min at +4 ^o^C at 14,000 rpm to pellet any possible white blood cell (WBC) contaminant from the buffy coat. Plasma fractions were then transferred to a clean tube and stored at -80 ^o^C for further analysis.

### Plasma cfDNA extraction and quantification

The cfDNA was extracted from one milliliter of plasma using the QIAamp Circulating Nucleic Acid Kit (Qiagen, Cat# 55114) as previously described [31] and was eluted in 35 μl elution buffer and stored at -20 ^o^C until further use. Plasma cfDNA concentration (ng/ml) was measured by Qubit Fluorometer using Qubit dsDNA HS Assay Kit (Life Technologies, Q32851). cfDNA copy numbers (copies/ml) was measured by real-time PCR targeting the human long interspersed nuclear element-1 (LINE1) sequences.

### Estimating cfDNA copy numbers by quantitative real-time PCR

Plasma cfDNA was quantified by quantitative real-time PCR using two sets of primers specific to the LINE1 sequences generating two distinct (79 bp and 148 bp) fragments following the protocol as previously described with slight modifications [32]. Briefly, samples’ copy numbers were estimated from a standard curve generated using a four 10-fold-dilution series of intact gDNA with known concentrations (100, 10, 1, and 0.1 pg/μl). First, primer pairs were used in regular PCR amplifications to confirm their target specificities of LINE1 chromosomal locations. PCR products were visualized by agarose gel electrophoresis for the expected size fragment and for any nonspecific bands as well as primer dimers. The primers were then used in the quantitative real-time PCR reactions. Additionally, at the end of each real-time PCR, melt curves were generated to further rule out any signal due to primer dimers. Real-time PCR reactions included SYBR green master mix and specific primers for each size fragment and a total amount of about 10 to 80 pg of sample cfDNA template in a 20 μl reaction volume. Each sample was run in triplicates to reduce pipetting errors when adding the sample DNA. Primer sequences and detailed real-time PCR protocol are shown in S1 Table. Estimated ng/ml amounts for samples were converted to copy numbers based on the human genome equivalence (GE), in which one copy of the human genome is considered to be approximately three picograms of DNA.

### Genotypic verification of twin status

In addition to disclosure by volunteers or their guardians, the twin statuses (monozygotic/dizygotic) were verified by the length polymorphism at short tandem repeat (STR) sequence analysis using AmpFlSTR Identifier PCR Amplification Kit (Applied Biosystems, 4322288). Genomic DNA (gDNA) was isolated from WBCs (buffy coat) of monozygotic and dizygotic twins following standard protocols. All steps were performed following the manufacturer’s protocol. Briefly, 5 to 10 ng of gDNA of each individual twin was PCR amplified in a multiplex PCR setting using specific primer pairs that target 15 STR loci and Amelogenin gene locus. The products for each twin were evaluated in Applied Biosystems 3500x/Genetic Analyzer.

### Consistency of plasma cfDNA isolation and real-time PCR quantification

To verify both the consistency of the cfDNA isolation from plasma and the real-time PCR quantification processes, a blood sample was collected from an individual and divided into four equal volumes, four separate plasma isolations and four independent cfDNA extractions were performed and then copy numbers for each cfDNA sample was quantified in two independent real-time PCR experiments in two separate days (S1 Fig).

### Statistical analysis

All statistical analysis and figure preparations were carried out using R-Studio (version 3.5.1) with the gglot2 library package. Two sample t-tests (unpaired) were used to compare between continuous variables. One sample t-test was used to calculate CI. With the data that does not have a normal distribution Wilcoxon rank sum test was used. Various associations were determined by calculating Pearson’s correlation coefficient (r) between cfDNA values of monozygotic twins, dizygotic twins, and sibling pairs. A p-value <0.05 was considered significant. The r-values ≥ 0.90 were considered as very high correlations, between ≤ 0.70 to 0.90 high, ≤ 0.50 to 0.70 moderate, ≤ 0.30 to 0.50 low, and < 0.30 little or no correlation. The proportion of the shared genetic variance (A), common environmental variance (C), and non-shared environmental variance (E) was estimated by structural equation modeling (SEM) using *R* software with the lavaan library package based on the assumption that MZ twins share 100% of the genetic and 100% of the common environment as *rMZ* = 1(*A* + *C*) while DZ twins share 50% of the genetic and 100% of the environment as *rDZ* = 0.5(*A* + *C*). The estimates for variant components are reported as percent values. The data used in analysis and figure preparations can be found in Supporting Information (S1 Data).

## Results

### Plasma cfDNA is significantly elevated in cancer patients

**Fig 2:**
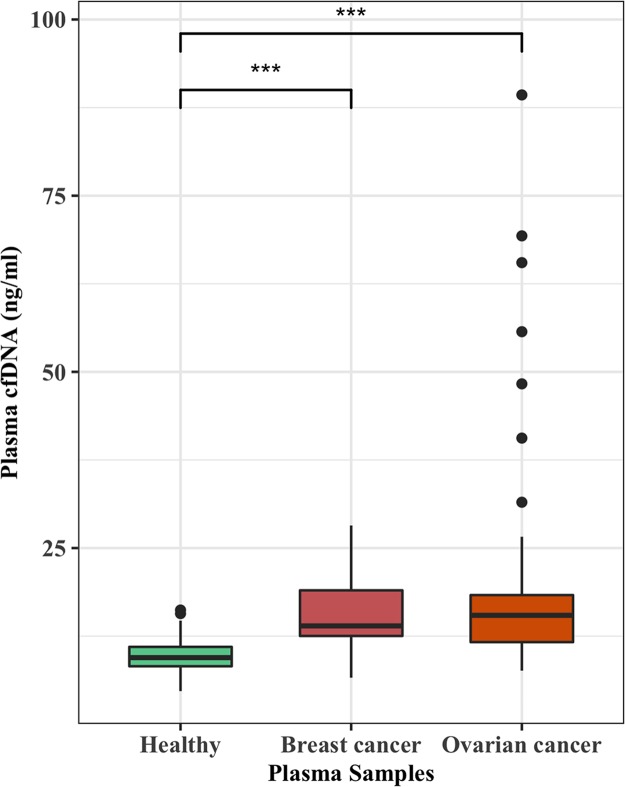
Plasma cfDNA levels are significantly elevated in cancer patients. Plasma cell-free DNA (cfDNA) was isolated from breast (n = 26) and ovarian cancer (n = 64) patients with various stages and undergoing treatment. Female subjects (n = 50) from healthy individuals of a similar age were selected as controls for breast and ovarian cancer patients. Separate cfDNA comparisons wre made between healthy female controls and the cancers patients by Wilcoxon rank sum test. Highly significant differences (***) were observed between cfDNA levels measured by Qubit fluorometer (ng/ml) of healthy females and breast cancer patients (p = 6.278e-07) and healthy females and ovarian cancer patients (p = 2.708e-11).

To validate that plasma cfDNA is increased in cancer patients, we measured cfDNA isolated from the plasma of breast (n = 26) and ovarian cancer (n = 64) patients and compared with that of cancer-free females (n = 50). The p-values between healthy and breast or ovarian cancer patients were estimated by the Wilcoxon rank-sum test. Plasma cfDNA levels for healthy individuals, breast cancer patients, and ovarian cancer patients ranged from 4.7 to 16.2, 6.6 to 28, and 7.6 to 89.3 ng/ml with median values of 9.5, 13.9, and 15.5, respectively (Fig 2). Even though there were highly significant differences between the cfDNA of healthy and breast cancers (p-value = 6.278e-07) and healthy and ovarian cancers (p-value = 2.708e-11), on the individual levels there were many overlapping cfDNA values between cancer-free individuals and cancer patients.

### Higher concordances of cfDNA levels between individuals with similar genetic and environmental background

**Fig 3:**
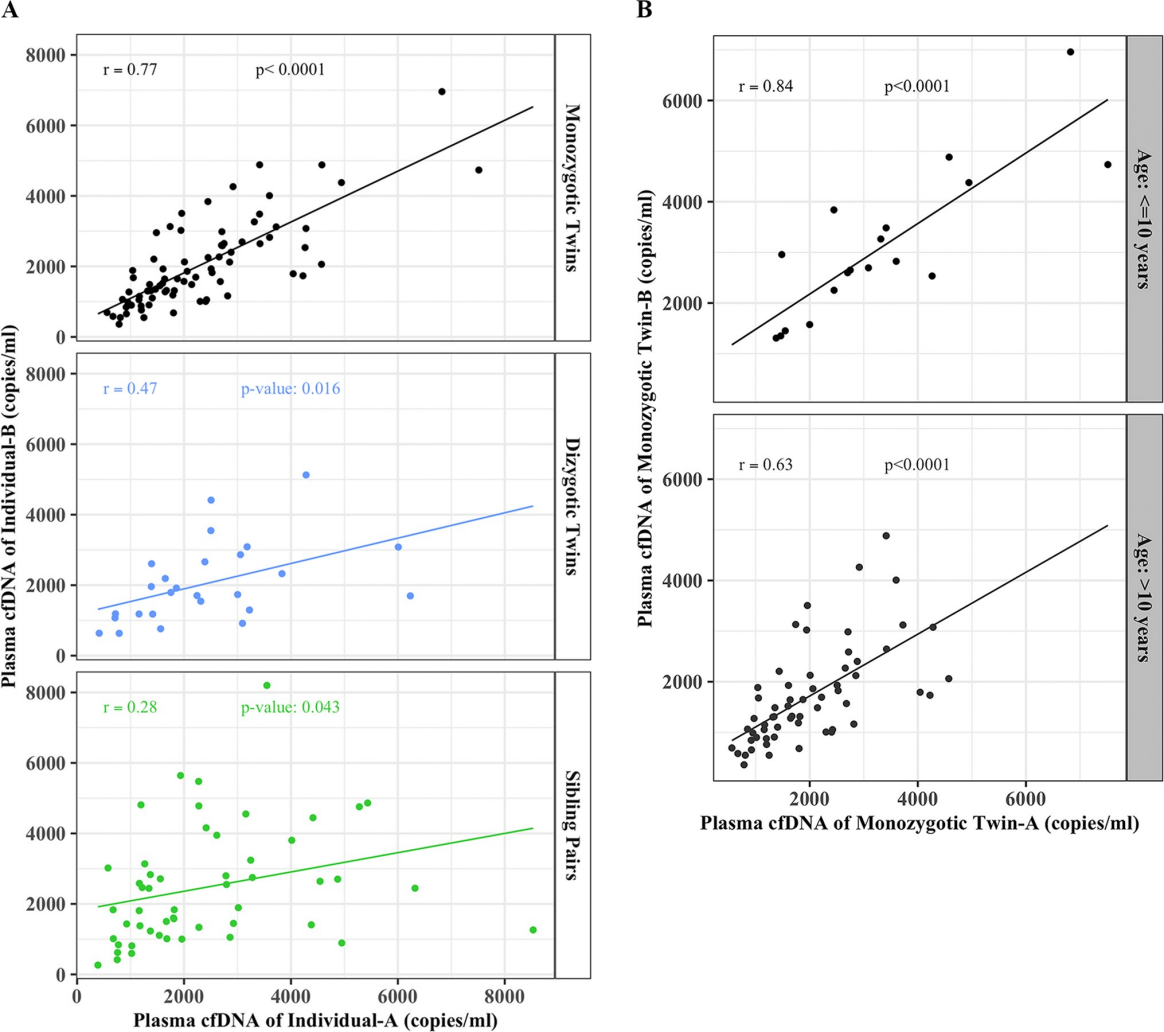
Higher concordances of cfDNA levels between individuals with similar genetic and environmental backgrounds. Scatter plots illustrating associations (Pearson’s correlation coefficients) between plasma cfDNA copy numbers (copies/ml) measured by real-time PCR for both 79 bp and 148 bp fragments of; (**A)** monozygotic twins (top panel) (r = 0.77, 95% CI = 0.66–0.85; p < 2.2e-16), dizygotic twins (middle panel) (r = 0.47, 95% CI = 0.10–0.74; p = 0.016), and siblings (bottom panel) (r = 0.28, 95% CI = 0.01–0.52; p = 0.04); (**B)** monozygotic twins with age ≤ 10 years (r = 0.84, 95% CI = 0.62–0.94; *p* = 1.178e-05) and >10 years-old (r = 0.63, 95% CI = 0.44–0.76; *p* = 8.622e-08).

To find the degree of associations between the cfDNA levels of individual pairs (monozygotic, dizygotic, and siblings), we performed the Pearson’s correlation coefficient test. We found a high correlation between plasma cfDNA levels (copies/ml) of monozygotic twins with a Pearson’s correlation coefficient of (r) = 0.77 with 95% confidence interval (CI) of 0.66 and 0.85 and a p-value < 2.2e-16 (Fig 3A). However, this high association was gradually reduced to moderate between dizygotic twins (r = 0.47, CI, 0.1–0.72; p-value = 0.016) and low association between sibling pairs (r = 0.28 CI, 0.0–0.52; p = 0.04) (Fig 3A). When we further stratified the monozygotic twins into two separate groups based on their age; ≤ 10 years old and >10 years old, we found an even higher association between twins of the younger age (r = 0.84, CI, 0.62–0.94, p = 1.178e-05) as compared to older ones (r = 0.62; CI, 0.44–0.76; p = 8.622e-08) (Fig 3B).

In order to quantify the genetic and the environmental components contributing to the variation in plasma cfDNA levels among individuals, we used ACE model (A = additive genetic factors, C = common or shared environmental factors, E = unshared or specific environmental factors).

Even though with limited sample size of MZ and DZ twin pairs, the ACE model analysis results indicated that plasma cfDNA levels are influenced by both genetic and environmental factors in similar proportions, 53% and 47% respectively. Each ACE component was accounted for A as 53%, C as 22.5%, and E as 24.5% of the variation in plasma cfDNA levels.

The actual copy number values for all twins and sibling pairs for both fragments (79 bp and 148 bp) are illustrated in S2 Fig.

### Substantial variation in plasma cfDNA levels among healthy individuals

**Fig 4:**
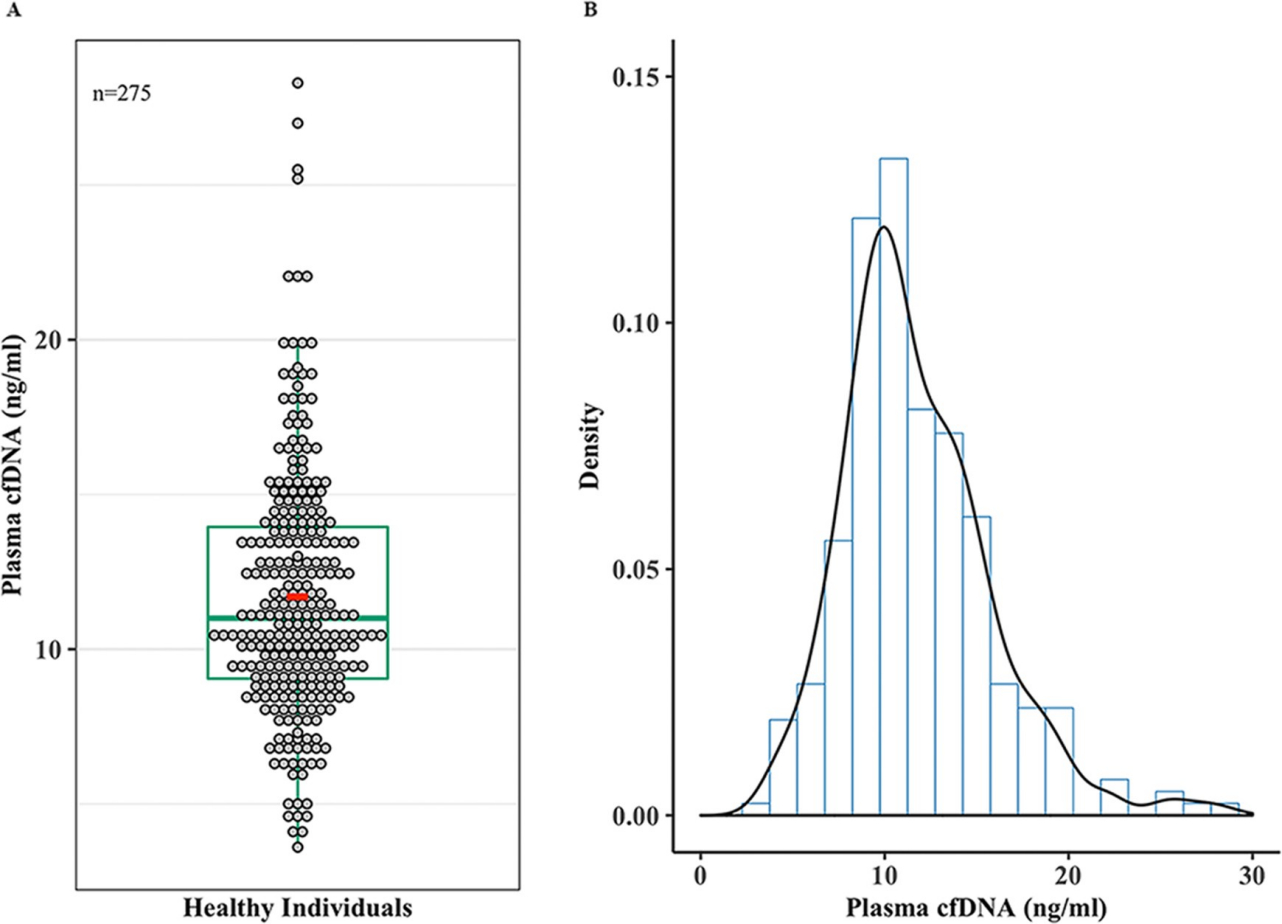
Substantial variation in plasma cfDNA levels among healthy individuals. (**A)** Dot plot overlaid by a box plot showing plasma cfDNA concentrations measured by Qubit fluorometer in nanograms (ng/ml) for all the samples (n = 275). Each circle represents a cfDNA value from an individual and 1^st^, second (median), and 3^rd^ quartiles are indicated on box plot, red circle shows the mean. **(B)** A density curve showing the overall distribution of plasma cfDNA concentrations (ng/ml) for the healthy individuals.

We determined the mean, median, CI, and overall distribution of cfDNA levels in disease-free individuals. The overall cfDNA levels in our disease-free individuals ranged from 3.6 to 28.3 ng/ml with the 1^st^, 2^nd^ (median), and 3^rd^ quartiles being 9.05, 11.0, and 13.05, respectively. The mean cfDNA value for the healthy individuals was 11.7 ng/ml with 95% CI of 11.2 and 12.2 (Fig 4A). Furthermore, the overall cfDNA levels across our healthy population displayed a normal distribution (Fig 4B).

### Plasma cfDNA is highly degraded

**Fig 5:**
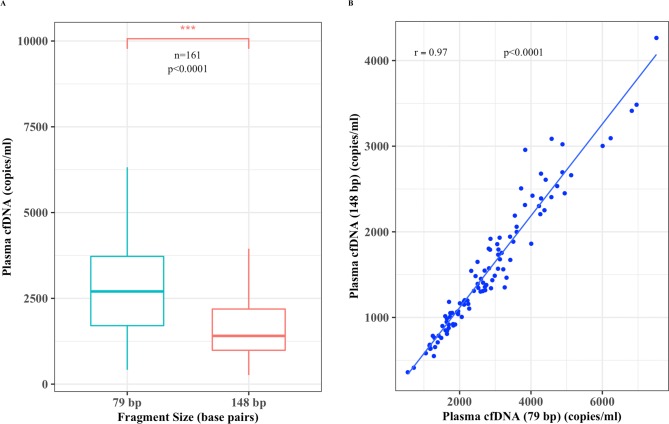
Plasma cfDNA is highly degraded. **(A)** Box blot displaying sample plasma cfDNA copies measured by real-time PCR (copies/ml) median; vertical bar, 2701 and 1406) (n = 161) for both 79 bp and 148 bp fragments, respectively and **(B)** a scatter plot showing a positive linear correlation (r = 0.97, 95%CI = 0.95–0.98, p < 2.2e-16) between the copy numbers of both fragments.

To also see the degree of degradation and association between the two different cfDNA size fragments (79 bp and 148 bp), Pearson’s correlation coefficient test was performed. We detected relatively higher plasma cfDNA copy numbers (p < 2.2e-16) of the shorter size fragment (79 bp vs. 148 bp) (Fig 5A). The overall total cfDNA copy numbers ranged from 417 to 8531 for the 79 bp fragment and 264 to 5643 for the 148 bp size fragment. The median and mean values for the 79 and 148 bp fragments were 2701 and 2912, 1406 and 1655 copies per ml of plasma, respectively. Furthermore, there was a very high correlation (r = 0.97; CI, 0.95–0.98; p < 2.2e-16) between total copy numbers of both fragments for each individual that further verifies the sensitivity of the real-time PCR quantification assay (Fig 5B).

### Higher concordance between twins and sibling pairs in cfDNA degradation status

**Fig 6:**
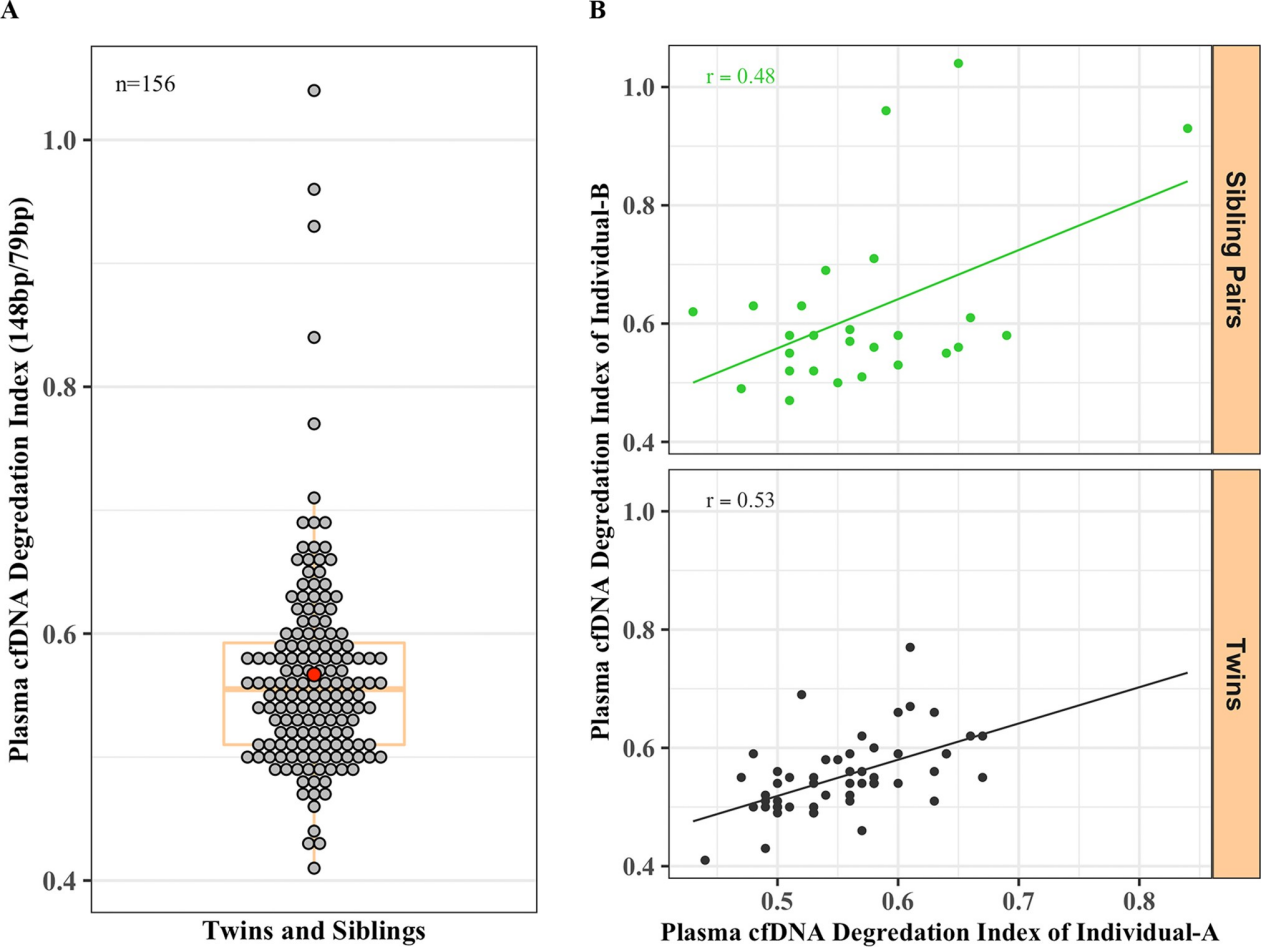
Higher concordance between twins and sibling pairs in cfDNA degradation status. (**A)** The overall range in cfDNA degradation index (total copy numbers of 148 bp fragment / total copy numbers of 79 bp) (mean = 0.57, 95% CI, 0.55–0.58) and (**B)** the association between the degradation indices of sibling pairs (upper panel, r = 0.48, CI, 0.11–0.73, 0.012) and all the twins (both monozygotic and dizygotic) (lower panel, r = 0.53, CI, 0.30–0.70, p = 4.548e-05).

In addition to plasma cfDNA levels, we also analyzed the cfDNA degradation status of twins and sibling pairs. The cfDNA degradation indices for all twins and siblings were calculated by dividing the total copy numbers of the 148 bp size fragment by the total copy numbers of the 79 bp size fragment for each individual. The overall degradation index ranged from 0.41 to 1.04, 1^st^, median, and 3^rd^ quartile of 0.51, 0.56, and 0.59, respectively. The mean degradation index for both twins and siblings was 0.57 with the 95% CI of 0.55 and 0.58 (Fig 6A). We also observed relatively similar cfDNA degradation indices between sibling pairs (r = 0.48; CI, 0.11–0.73; p = 0.013) and twins (r = 0.53, CI, 0.31–0.70, p = 4.548e-05) (Fig 6B).

### While gender influences cfDNA levels, the age has no effect

**Fig 7:**
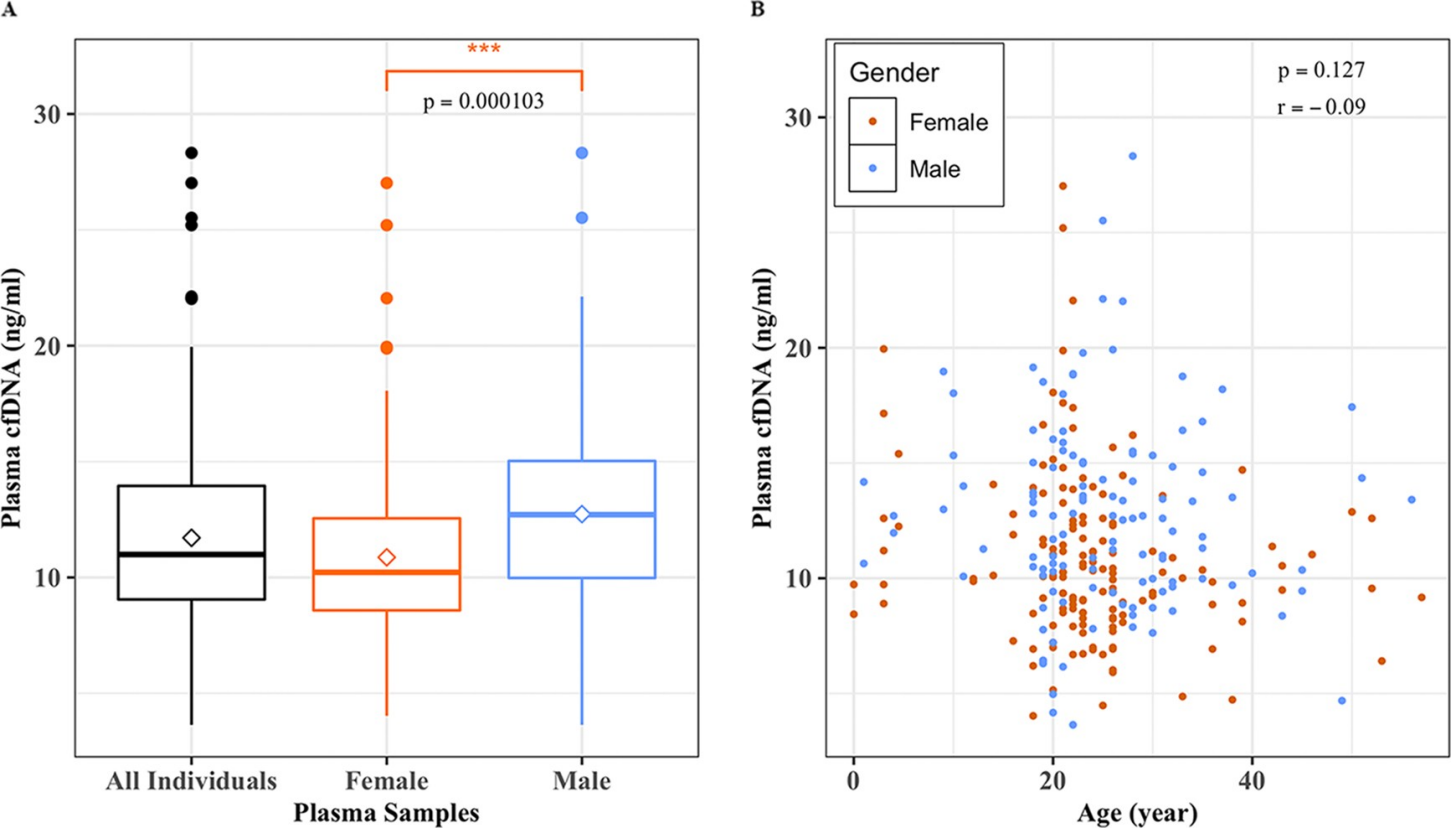
While gender influences cfDNA levels, the age of the individuals has no effect. (**A)** A box plots showing mean (diamond shape) and median (vertical bar) plasma cfDNA levels measured by Qubit fluorometer (ng/ml) for all samples (n = 275), female (n = 151; mean = 10.9 ng/ml), and male (n = 124, mean = 12.7 ng/ml). Two sample t-test shows that there is a highly significant difference between female and male subjects (p = 0.0001) in cell-free DNA levels (ng/ml); **(B**) A scatter plot showing no association between age and plasma cfDNA levels (ng/ml).

We analyzed the cfDNA levels to find whether gender and/or age of the individual had any effect on cfDNA levels. Two sample t-test showed an overall higher plasma cfDNA level (ng/ml) in males as compared to that of females (p-value = 0.0001) (Fig 7A). The mean plasma cfDNA levels for females and males were 10.9 and 12.7 ng/ml, respectively. On the other hand, our results indicated that there was no apparent correlation between the age of the individual and plasma cfDNA concentration (ng/ml) irrespective of the gender (r = -0.09, CI, -0.21–0.027; p = 0.127) (Fig 7B). The overall age of our disease-free population ranged from 0 to 57 years with a median age of 23 years.

### Every family has its own unique cfDNA level

**Fig 8:**
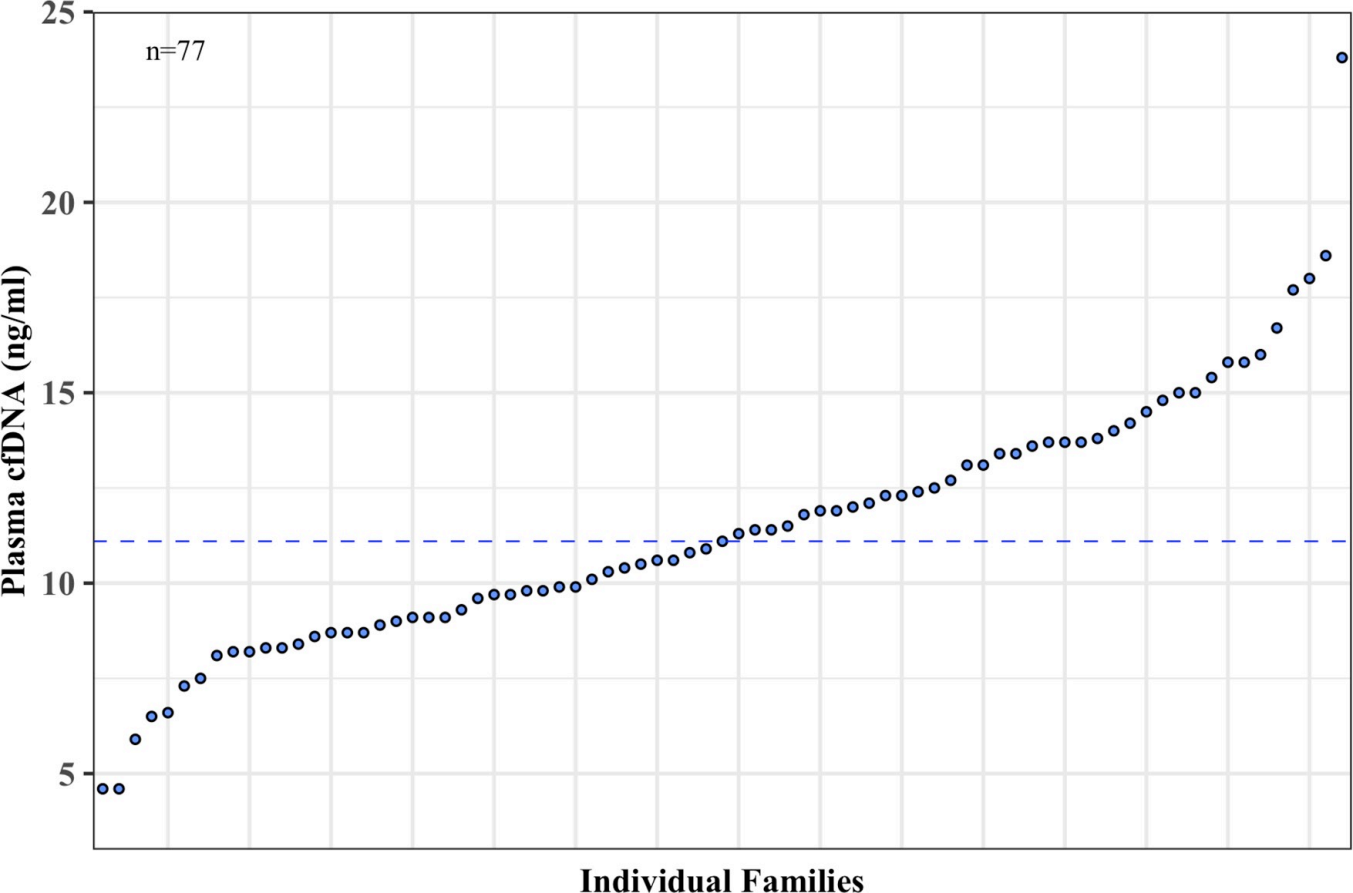
Each family has its own unique cfDNA level. The mean plasma cell-free (cfDNA) concentration (ng/ml) measured by Qubit fluorometer for individual families (n = 77) is shown in a scatter plot where each dot represents the mean cfDNA value for a single family. The median plasma cfDNA level (11.1 ng/ml) is shown by a dotted line that separates the families into two main groups; families with mean cfDNA levels below and above 11.1 ng/ml.

Similar to the variation among individuals, we also found an inherent variation among families. We quantified the plasma cfDNA collected from a total of 77 separate families each of which had at least two members of twins and/or siblings (Fig 8). The overall cfDNA levels ranged from 4.6 to 23.8 ng/ml with a median value of 11.1 ng/ml and a mean value of 11.4 ng/ml with 95% CI of 10.7 and 12.2 ng/ml. Families appeared to fall into two main groups based on their median plasma cfDNA levels (< 11.1 and > 11.1 ng/ml).

## Discussion

As current therapies have been shifting towards personalized and precision modalities with consideration of single person clinical trials for better efficacies [33–35], similar approaches in disease detection should be considered for increased specificity and sensitivity.

In this proof-of-principle study, we attempted to understand the relative contribution of genetic and environmental factors on the levels of plasma cfDNA and possible use as a personalized biomarker for disease detection. We therefore analyzed cfDNA from a cohort that included twins, sibling pairs, genetically unrelated healthy individuals, and cancer patients (Fig 1). Our findings are summarized in the following main points; first, similarities/differences in both the individual’s genotype and environment (53% and 47%) have a high impact in defining the ultimate levels of plasma cfDNA; second, there is a substantial variation in cfDNA levels among healthy individuals presenting an approximately normal distribution; third, while there is a significant difference in cfDNA levels based on the gender, age appeared to have no influence on individuals’ cfDNA levels; lastly, even though there is a slight variation among family members, every family has an inherent plasma cfDNA level.

As reported in most previous studies [20,22,36,37], we also observed elevated cfDNA levels in breast and ovarian cancer patients compared to cancer-free individuals however there were substantial overlaps in cfDNA levels between individuals of the two separate groups. Even though our results showed a highly significant increase in cfDNA levels of cancer patients, variations in cfDNA levels even among healthy individuals make the simple evaluation of cfDNA unreliable to be used as a biomarker for cancer detection on the individual level due to many overlapping values between cancer and cancer-free individuals.

To our best knowledge, our study is the first to evaluate cfDNA levels in monozygotic twins, dizygotic twins, and sibling pairs. Our results show that cfDNA levels appear to be highly correlated in genetically and environmentally identical individuals (monozygotic twins) and the correlation gradually decreases as the genetic and environmental similarities decrease in dizygotic twins, sibling pairs, and in unrelated individuals. Historically, classic twin studies have significantly contributed to our understanding of the genetic and environmental basis of various syndromes and diseases due to the same genetic make-up of monozygotic twins [38,39]. It is suggested that not only does an individual get its genome from its parents, but also inherits the environment [40,41], which could be the mechanism influencing the similarities or differences in cfDNA levels in our study population. The data presented here clearly indicates that the slight variation in plasma cfDNA levels even between monozygotic twins is possibly due to differences in environmental exposures. This suggests that both the genetic makeup and the environment collectively modulate the cfDNA level of any given individual.

Our data also showed a substantial variation in cfDNA levels across disease-free individuals displaying a normal distribution. This is not the first study determining the variation in plasma cfDNA levels however it is the only one evaluating cfDNA in healthy pairs who had varying degrees of genetic and environmental backgrounds. The variation in cfDNA levels among healthy individuals has been reported only as control groups in cancer studies [2,20]. However, these bulk comparisons are not always accurate as there are overlapping cfDNA levels between healthy individuals and patients, thus rendering cfDNA evaluation at disease onset alone unreliable for use as a non-invasive biomarker [19]. Furthermore, the overall elevation in plasma cfDNA levels is reported to be significant mostly in late stage cancers therefore diminishing its predictive use as a biomarker as most other diagnostic modalities can easily detect late stage cancers. Moreover, using the cfDNA levels from healthy individuals as controls for patients whom are genetically unrelated could be one of the reasons for the discordant results between studies [36,42].

Our results showed significant differences in cfDNA levels between genders; males tend to have higher plasma cfDNA levels than that of females. There are no previous reports correlating cfDNA levels with the gender of healthy individuals. The differences found in plasma cfDNA concentrations between males and females suggest that gender should be taken into account when making both diseased and control group comparisons. Our study however showed that the age of the individual does not have an effect on plasma cfDNA levels. To our knowledge, there are no studies showing the effect of age on cfDNA levels except in a recent study that evaluated age related epigenetic changes using plasma cfDNA [43]. Furthermore, even though there are some differences in cfDNA levels among genetically related siblings, every family seems to have an inherent plasma cfDNA level. To date, there are no reports evaluating plasma cfDNA in families.

In conclusion, our study demonstrated that both genomic and environmental factors modulate the individual’s cfDNA level and is therefore highly variable in the healthy population. Our findings suggest that the diagnostic sensitivity of cfDNA evaluation as a non-invasive biomarker could be improved if the person’s cfDNA level is known prior to disease onset or cancer presentation. If further verified in larger cohorts, plasma cfDNA levels could thus serve as a sensitive non-invasive personalized biomarker for diagnosis and prognosis of many diseases, particularly cancers.

## Supporting information

S1 Data Values used in statistical analysis and figure generations. (XLSX)

S1 Fig Sample to sample consistency of cell-free DNA (cfDNA) extraction and day-to-day real-time PCR quantifications. The mean ± SE plasma copy numbers (copy/ml) of cfDNA isolated from an individual blood sample in four independent extractions and quantified by two separate real-time PCR reactions in two consequent days (day-1 and day-2) for both 79 bp and 148 bp size fragments. Day-1 (2257 ± 65 vs. 1253 ± 74) and Day-2 (2274 ± 56 vs. 1251 ± 66). There was no statistically significant difference between four independent plasma isolations and cfDNA extractions and also no day-to-day variation in separate real-time PCR quantifications (p < 0.0001).
(TIF)

S2 Fig Plasma cell-free DNA (cfDNA) of 79 bp and 148 bp fragments measured by real-time PCR (copies/ml) for all; (**A**) monozygotic twins (n = 39), **(B**) dizygotic twins (n = 13), and (**C**) sibling pairs (n = 26). Each circle represents the plasma copy number for each individual sample and size fragments are filled with separate colors as shown.
(TIF)

S1 Table The detailed real-time PCR protocol that includes primer sequences, reaction mix and reaction settings used throughout the study. (DOCX)
