# Supplementary figures and images for: Cell-free DNA levels of twins and sibling pairs indicate individuality and possible use as a personalized biomarker
Source: PLoS One. 2019 Oct 10;14(10):e0223470. doi: 10.1371/journal.pone.0223470 (PMC6786590; doi:10.1371/journal.pone.0223470)

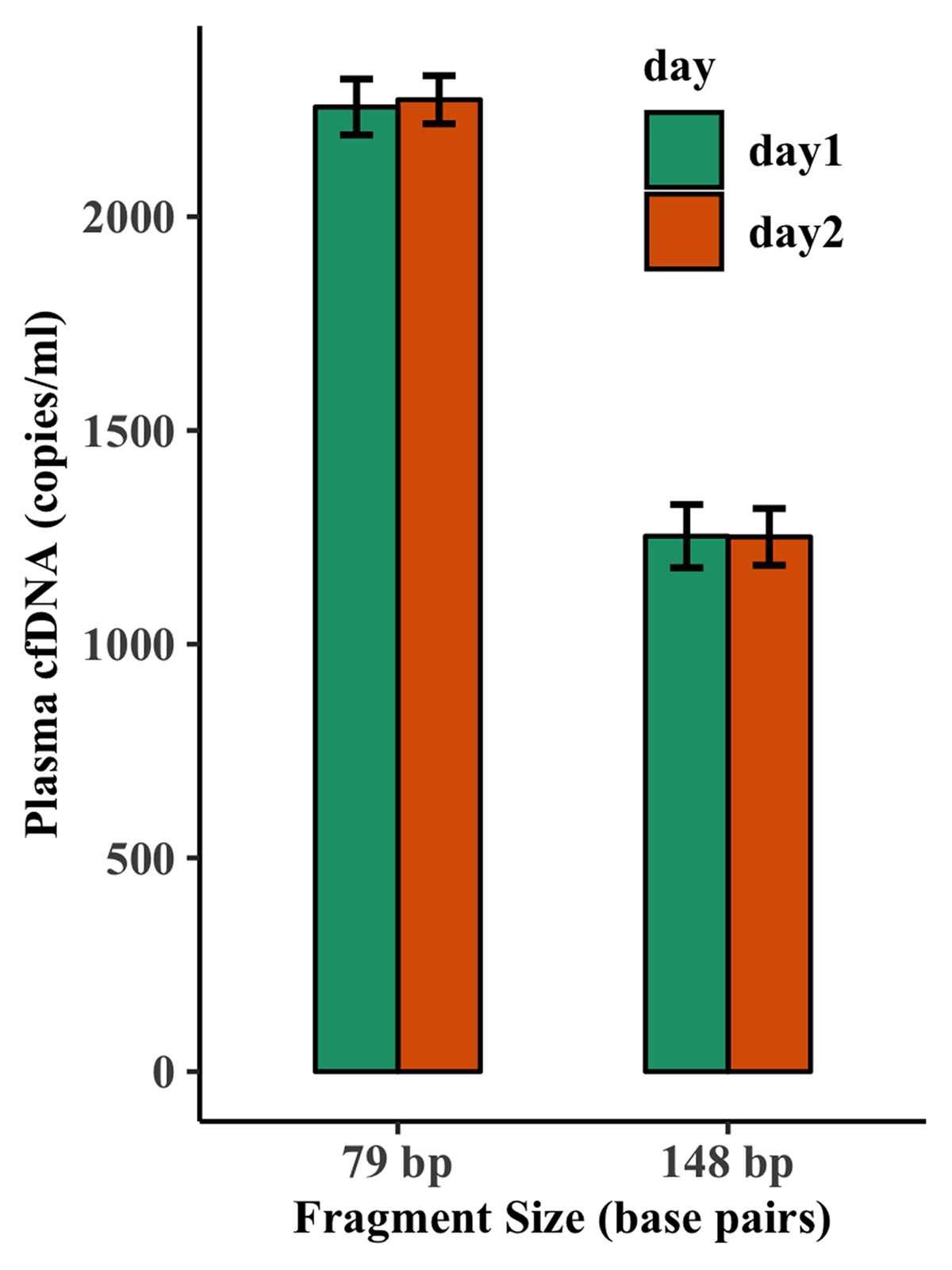

Supplement: S1 Fig — The mean ± SE plasma copy numbers (copy/ml) of cfDNA isolated from an individual blood sample in four independent extractions and quantified by two separate real-time PCR reactions in two consequent days (day-1 and day-2) for both 79 bp and 148 bp size fragments. Day-1 (2257 ± 65 vs. 1253 ± 74) and Day-2 (2274 ± 56 vs. 1251 ± 66). There was no statistically significant difference between four independent plasma isolations and cfDNA extractions and also no day-to-day variation in separate real-time PCR quantifications (p < 0.0001). (TIF) [file pone.0223470.s002.tif]

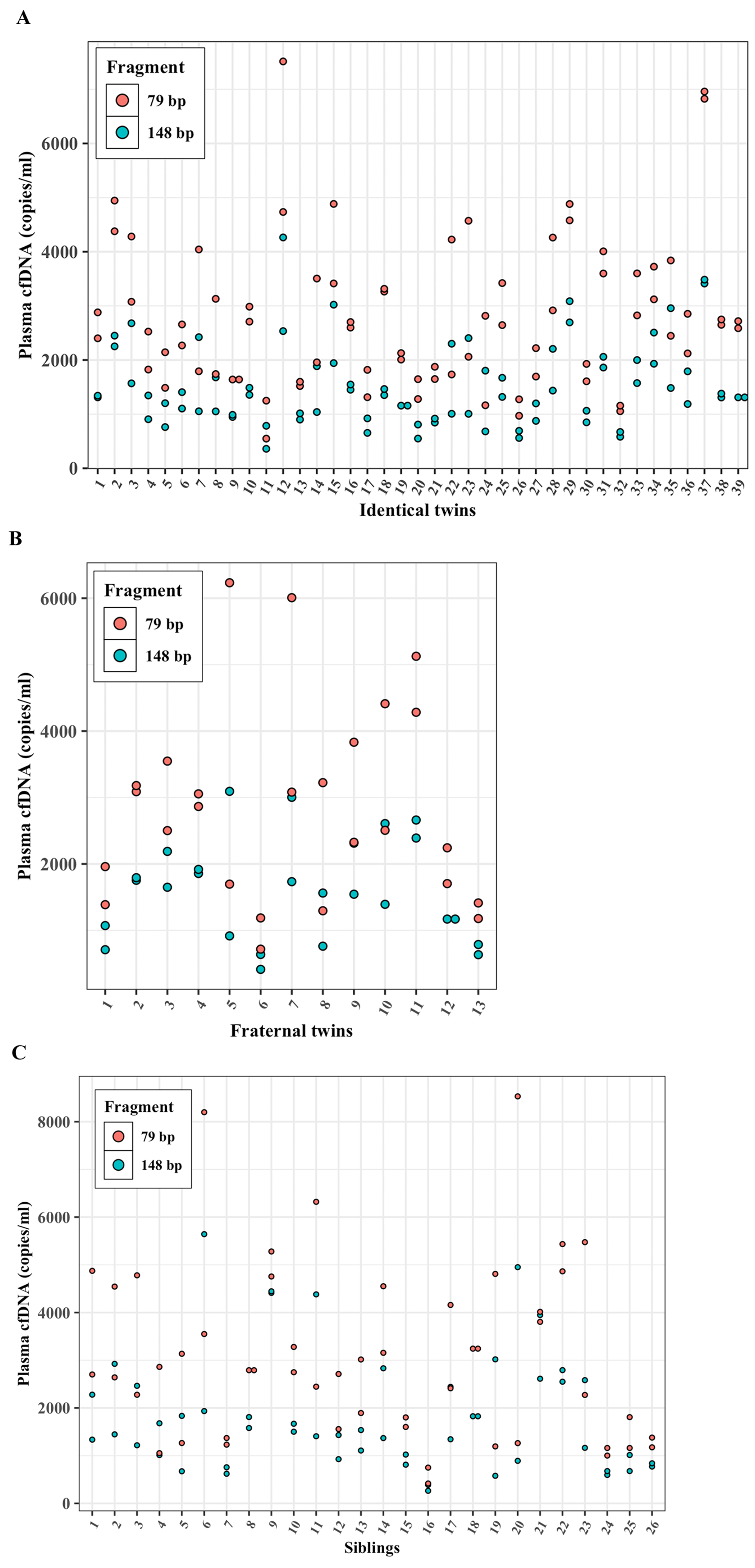

Supplement: S2 Fig — Plasma cell-free DNA (cfDNA) of 79 bp and 148 bp fragments measured by real-time PCR (copies/ml) for all; (A) monozygotic twins (n = 39), (B) dizygotic twins (n = 13), and (C) sibling pairs (n = 26). Each circle represents the plasma copy number for each individual sample and size fragments are filled with separate colors as shown. (TIF) [file pone.0223470.s003.tif]
