# Supplementary material for: Cell-free DNA levels of twins and sibling pairs indicate individuality and possible use as a personalized biomarker
Source: PLoS One. 2019 Oct 10;14(10):e0223470. doi: 10.1371/journal.pone.0223470 (PMC6786590; doi:10.1371/journal.pone.0223470)
Supplement: S1 Table — (DOCX) [file pone.0223470.s004.docx]

| **LINE1 Specific Primers** | | | | | |
| --- | --- | --- | --- | --- | --- |
| **Primer** | | **Sequence (5'**−**>3')** | | **Amplicon Size (bp)** | |
| LINE1-79-Forward | | AGGGACATGGATGAAATTGG | | 79 | |
| LINE1-79-Reverse | | TGAGAATATGCGGTGTTTGG | |  |  |
| LINE1-148-Forward | | ACTTGGAACCAACCCAAATG | | 148 | |
| LINE1-148-Reverse | | TGAGAATGATGATTTCCAATTTC | |  |  |
| **Real-time PCR Reaction Mix** | | | | | |
| **Reagent** | **Volume (μl)** | | **Final Concentration / Total amount** | | |
| Master Mix (2X)^a^ | 10 | | 1X | | |
| LINE1 specific primer mix (20 μM) | 0.3 | | 0.015 μM | | |
| DNAse-free water | 7.7 | | N/A | | |
| gDNA/cfDNA^b^ | 2 | | 0.2 to 200 pg / 10 to 80 pg | | |
| **Total** | **20** | | - | | |
| **Thermocycler Reaction Settings** | | | | | |
| **Step** | | **Enzyme activation** | **Real-time PCR** | | |
|  |  |  | Cycles (32X) | | |
|  |  |  | Denaturation | | Annealing/Extension |
| Time | | 10 min | 15 sec | | 1 min |
| Temperature (^o^C) | | 95 | 95 | | 60 |

**S1 Table.**
